# Supplementary material for: MalariaSED: a deep learning framework to decipher the regulatory contributions of noncoding variants in malaria parasites
Source: Genome Biol. 2023 Oct 16;24:231. doi: 10.1186/s13059-023-03063-z (PMC10577899; doi:10.1186/s13059-023-03063-z)
Supplement: Supplementary file 2 — Additional file 2: Fig. S1. Evaluating the performance of MalariaSED using different lengths of DNA sequence input. Fig. S2. TFs binding effects of single nucleotide substitutions at 5-mers sequences predicted by MalariaSED in P. falciparum. Fig. S3. MalariaSED prediction for epigenetic markers of ~1.3 million variants gathered by Pf3K. Fig. S4. MalariaSED prediction results for de novo mutations at non-coding regions discovered from the single cell study. Fig. S5. The geographically differentiated variants accompanied by different chromatin profile effects in the noncoding region are more likely closer to genes with high levels of geographic differentiation. [file 13059_2023_3063_MOESM2_ESM.pdf]

Fig. S1

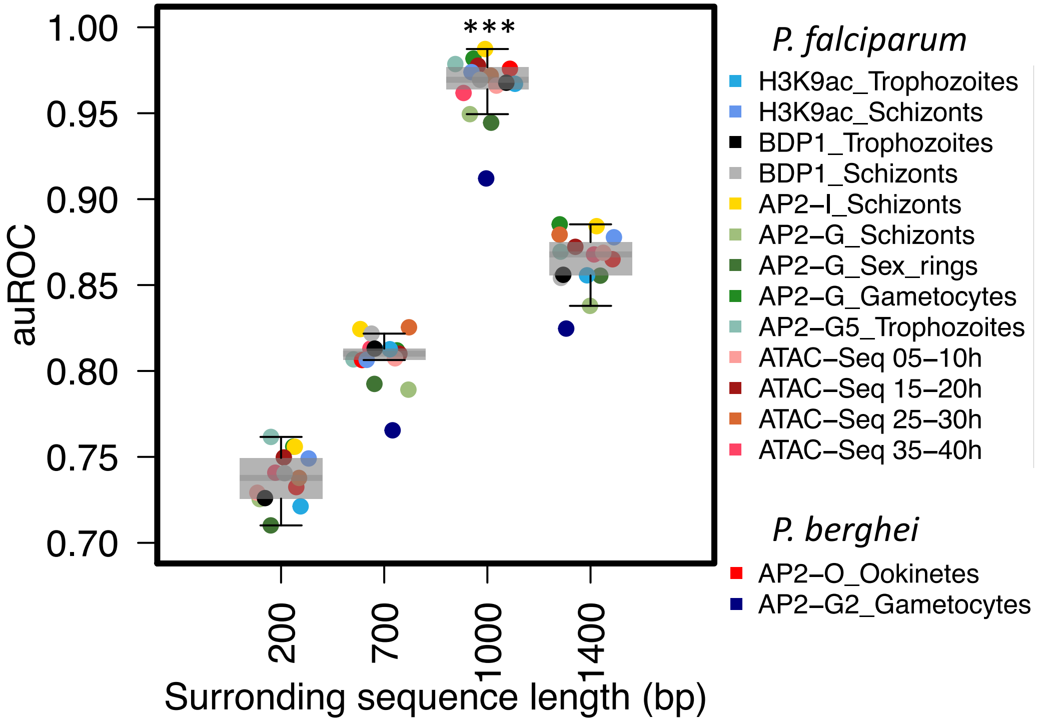

Fig. S1. Evaluating the performance of MalariaSED using different lengths of DNA sequence input. The same DL architecture as MalariaSED was trained on 200, 700, 1000 and 1400 bp DNA sequences, respectively. We present the auROC of each model as a point and use a box plot to show the distribution of the models inputting the same DNA sequence length. The results indicate 1000bp input outperforms other length input ('***' indicates Wilcoxon test $p < 0.01$ compared between the 1kb input sequence and any other length listed here).

Fig. S2

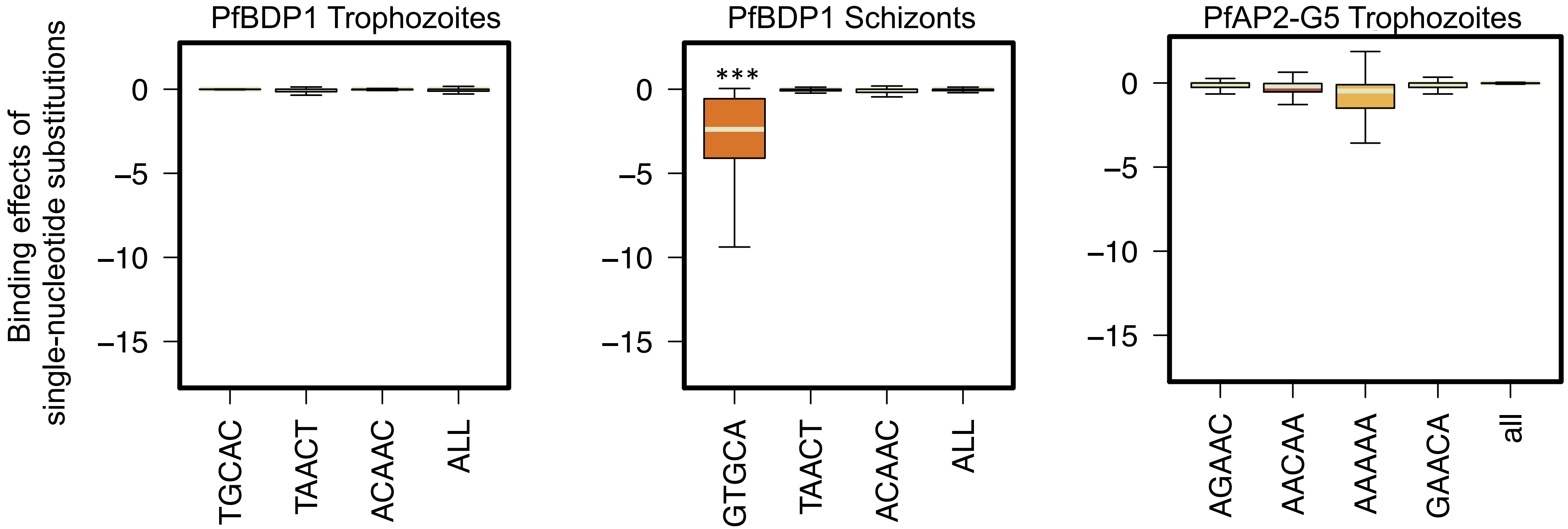

Fig. S2. TFs binding effects of single nucleotide substitutions at 5-mers sequences predicted by MalariaSED in *P. falciparum.* ‘***’ indicates Wilcoxon test compared with whole-genome background $p < 2.2e-16$.

Fig. S3

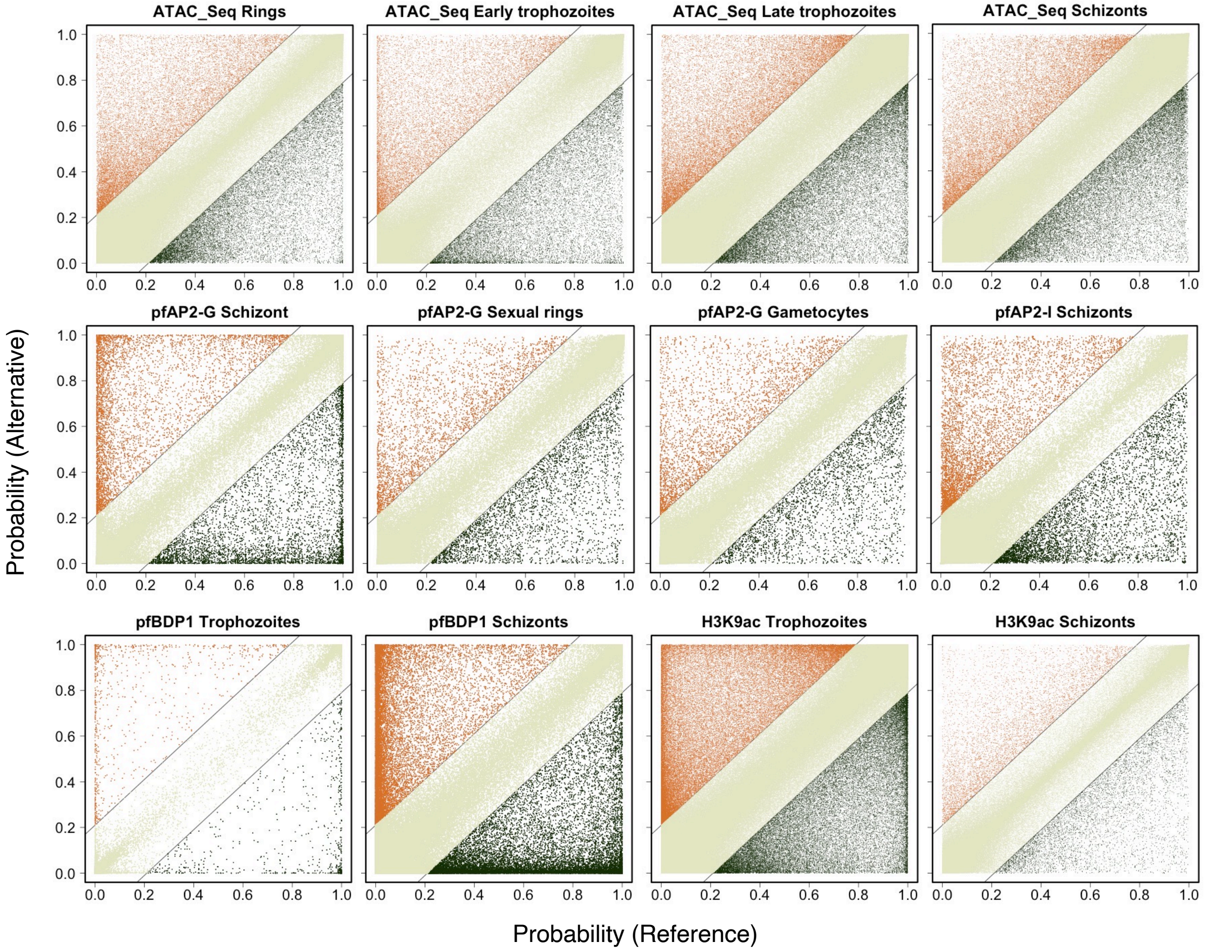

Fig. S3. MalariaSED prediction for epigenetic markers of ~1.3 millions variants gathered by Pf3K. The horizontal and vertical direction represent, respectively, the predicted probabilities that the sequences carrying the reference allele and alternative allele.

Fig. S4

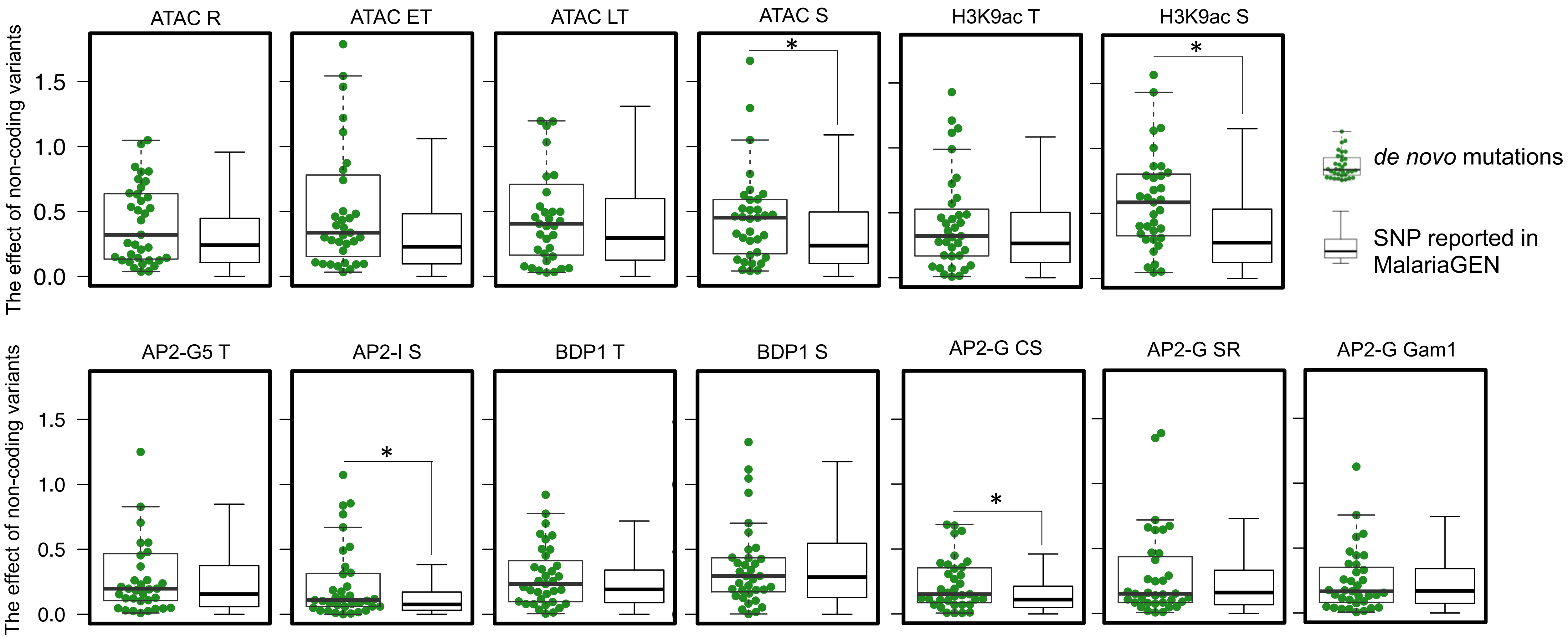

Fig. S4. The results from MalariaSED indicates de novo mutations at non-coding regions discovered from the single cell study have higher chance to alter their surrounding chromatin profiles in schizonts. '*' indicates Wilcoxon test compared with non-coding SNPs reported from Pf3K, $p < 0.05$.

Fig. S5

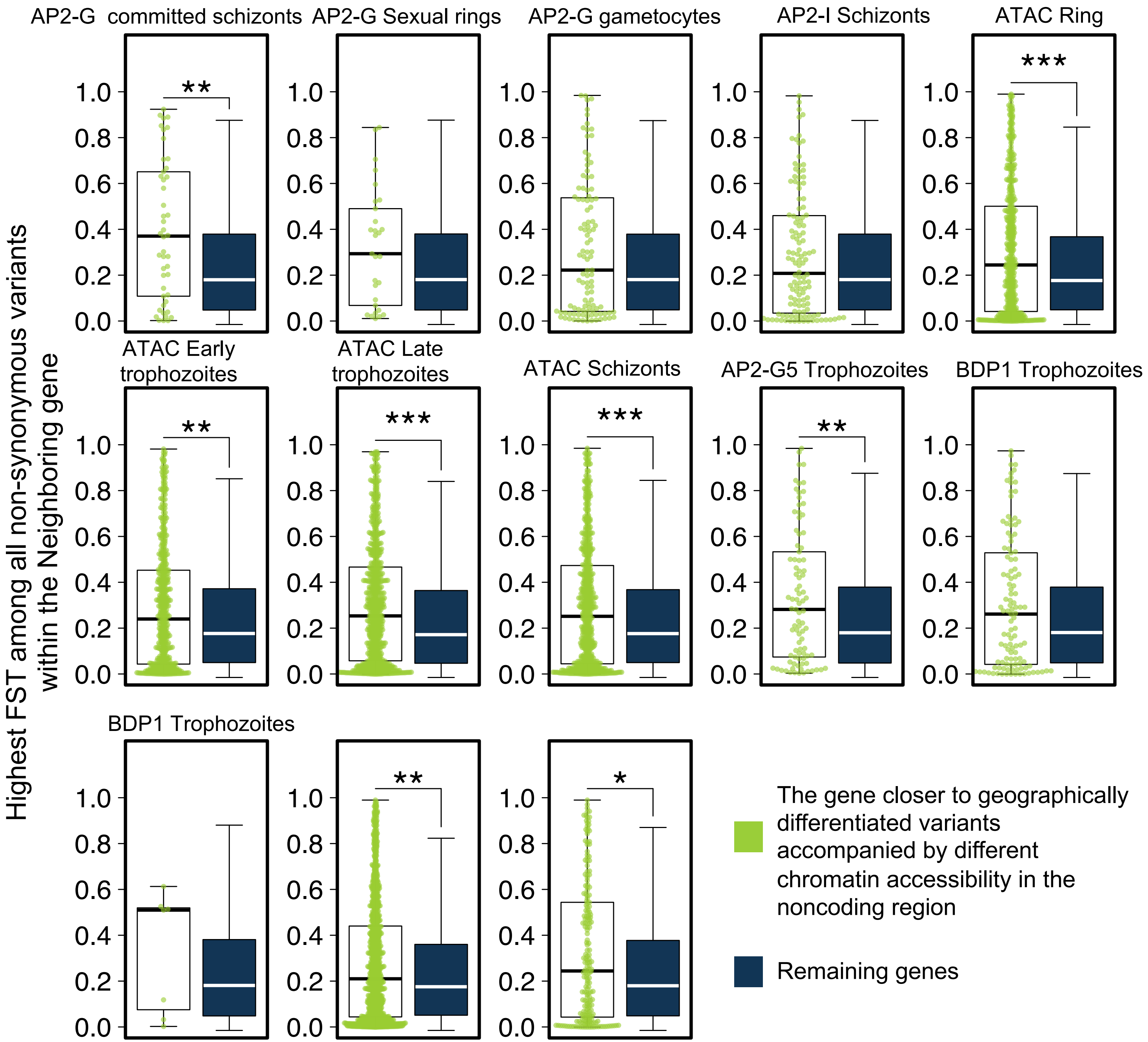

Fig. S5. The geographically differentiated variants accompanied by different chromatin profile effects in the noncoding region are more likely closer to genes with high levels of geographic differentiation. Each green dot represents a gene closest to noncoding variants presenting global FST > 0.1 and high chromatin effects (the top 1% of chromatin effects). '*' represents Wilcoxon test p< 0.05, '**' is p < 0.01, while '***' < 1e-3.
